# Knowledge Discovery in Spectral Data by Means of Complex Networks

**DOI:** 10.3390/metabo3010155

**Published:** 2013-03-11

**Authors:** Massimiliano Zanin, David Papo, José Luis González Solís, Juan Carlos Martínez Espinosa, Claudio Frausto-Reyes, Pascual Palomares Anda, Ricardo Sevilla-Escoboza, Stefano Boccaletti, Ernestina Menasalvas, Pedro Sousa

**Affiliations:** 1 Faculdade de Ciências e Tecnologia, Departamento de Engenharia Electrotécnica, Universidade Nova de Lisboa, Portugal; E-Mail: pas@fct.unl.pt (P.S.); 2 Centre for Biomedical Technology, Polytechnic University of Madrid Pozuelo de Alarcón, 28223 Madrid, Spain; E-Mails: papodav@gmail.com (D.P.); stefano.boccaletti@gmail.com (S.B.); ernestina.menasalvas@upm.es (E.M.); 3 Innaxis Foundation & Research Institute, José Ortega y Gasset 20, 28006, Madrid, Spain; 4 Biophysics and Biological Science Laboratory, Centro Universitario de los Lagos, Universidad de Guadalajara, 47460, Lagos de Moreno, Jalisco, Mexico; E-Mails: jluis0968@gmail.com (J.L.G.S.); jcmartineze@ipn.mx (J.C.M.E.); sevillaescoboza@gmail.com (R.S.-E.); rjaimes@culagos.udg.mx (R.J.-R.); 5 Biotechnology and Mechatronic Academy Instituto Politécnico Nacional-UPIIG, 36275, Silao de la Victoria, Guanajuato, Mexico; 6 Centro de Investigaciones en Óptica, A. C. 20200, Aguascalientes, Mexico; E-Mail: cfraus@cio.mx (C.F.-R.); 7 Hospital Regional de Alta Especialización del Bajío 37660, León, Gto., Mexico; E-Mail: ppalomares61@hotmail.com (P.P.A.)

**Keywords:** complex networks, data mining, spectroscopy, classification

## Abstract

In the last decade, *complex networks* have widely been applied to the study of many natural and man-made systems, and to the extraction of meaningful information from the interaction structures created by genes and proteins. Nevertheless, less attention has been devoted to metabonomics, due to the lack of a natural network representation of spectral data. Here we define a technique for reconstructing networks from spectral data sets, where nodes represent spectral bins, and pairs of them are connected when their intensities follow a pattern associated with a disease. The structural analysis of the resulting network can then be used to feed standard data-mining algorithms, for instance for the classification of new (unlabeled) subjects. Furthermore, we show how the structure of the network is resilient to the presence of external additive noise, and how it can be used to extract relevant knowledge about the development of the disease.

## 1. Introduction

Metabolomics, *i.e*., the study of chemical processes involving metabolites, and more specifically metabonomics (the study of metabolic changes under external perturbations), are promising approaches for the understanding of the fundamental nature of many diseases and the related metabolic responses. Since the pioneering works of Nicholson and coworkers [1,2], metabonomics has aimed at the extraction of information about the functions of the organism from biofluids and tissues, and at the rapid detection of biological dysfunctions; it has proven extremely valuable for both diagnostic and prognostic purposes. 

The main problems tackled within metabonomics are the detection, characterization and classification of external perturbations (including diseases, drugs, *etc*.). Related studies have usually exploited different statistical tools for multivariate data analysis, along with classical data mining techniques. Some of the most recurrent are principal component analysis, partial least square discriminant analysis, genetic algorithms, and neural networks [3,4,5]. Here, we propose a new approach to the problem, *i.e*., the use of a *complex network* representation of data as a pre-processing step. 

Networks [6,7,8] are very simple mathematical objects, constituted by a set of nodes connected by links. Due to their simplicity and generality, they have become an invaluable tool for the analysis of complex systems, *i.e*., systems composed of a large number of elements interacting in a non-linear fashion, leading to the appearance of global *emergent behaviors* [9]. The application of complex network theory to metabolomics is not new. Networks can be created by connecting pairs of metabolites through the reactions they participate in, and the resulting topology reveals information about the functioning of the metabolic system [10] and its evolution through time [11]. 

Beyond this structural representation, it has been shown that complex networks can be used to support data analysis tasks. For instance, in neuroscience studies, nodes may represent individual sensors detecting the electric or magnetic field generated by groups of neurons, and the links between them may indicate the presence of some kind of correlation between their activity [12]. Another successful example is represented by genetic networks, where nodes represent individual gene expressions, and node pairs are connected when some functional relationship is experimentally detected (see, for instance, [13,14]). Nevertheless, and to the best of our knowledge, complex networks have never been applied to data analysis in metabonomics. 

In this contribution, we propose the use of a complex network representation of spectral data as a preliminary step for a classification task. We first introduce and describe in Section 2 a method for network reconstruction that uses raw spectral data as input information. This technique, adapted from previous studies aimed at genetic network reconstruction [15,16], detects significant correlations on the spectral levels of both healthy subjects and subjects known to suffer from a given disease. By means of suitable data mining techniques, the resulting networks can then be used to train classification algorithms. In Section 3 the technique is applied to urine samples of people suffering from a kind of nephritis; it is shown how the structure of the network provides information about the abnormal elements of the sample, and how the analysis is stable against measurement noise. Section 4 reports on another application, involving analysis of blood samples of people suffering from leukemia; behind the classification task, it is shown how information about the progress of the disease can be extracted. Finally, in Section 5 some conclusions are presented.

## 2. Description of the Method

The information relative to each subject is codified by means of a network. Each node represents one of the available spectral measurements, or a bin representing a group of them, and the links between two nodes identify pairs of measurements that exhibit characteristics related to the disease. 

Mathematically, let us suppose that the initial data available are represented by *n* measurements (or bins) for each one of the *m* subject under study. Furthermore, *m_c_* and *m_d_* subjects are labeled as healthy (control) or patients, respectively, with *m_c_* + *m_d_* = *m*. For the sake of simplicity, we will denote by *C* (*D*) the *n* × *m_c_* (*n* × *m_d_*) matrix containing all the data associated to control subjects (patients), so that the element *c_i,j_* (*d_i,j_*) identifies the *j* measurement of control subject (patient) *i*. These two groups will act as training data. We also suppose that there is an additional subject, identified as *X*, for which no label is available: the aim of the network-based analysis is therefore the reconstruction of a network representation for this new subject, the extraction of the associated network characteristics, and finally, the correct classification of the subject into one of the two classes. 

The creation of a network representation requires analyzing whether we should create a link between each possible pairs of nodes. For each possible pair of bins, therefore, it is necessary to identify if its value follows two different models for control and disease subjects respectively. Here, we suppose that both models are a linear correlation between pairs of bins. Specifically, we linearly fit the values of the two bins (in what follows, *i* and *j*) for both groups of labeled subjects:

*c_•__,j_* = *αc_•__,i_* + *β* + *ε* *d_•__,j_* = *α'd_•__,i_* + *β'* + *ε'* (1)

**Figure 1:**
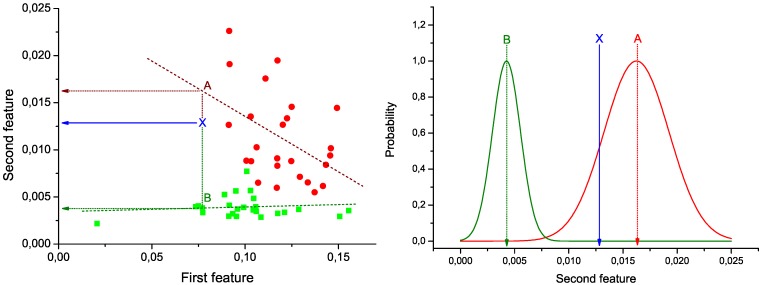
Example of calculation of the weight of a link. (Left) Lineal fit of data corresponding to control subjects and patients; (right) classification of an unlabeled subject (marked as *X*) into one of the two groups.

*α* and *α'* are the slopes of the two lineal fits (respectively, for the control and patient groups), *β* and *β'* the two intercepts, and *ε* and *ε'* two vectors with the residuals of the fits. This step is represented in Figure 1 (Left): green squares (red circles) represent the pair of values under analysis for control subjects (patients), and green and red dashed lines the best lineal fit for each group. Notice that these lines represent the expected behavior of the two bins under analysis in each group of data. Therefore, the problem of the classification of a new subject can be seen as the identification of the line to which its values are closer. 

In Figure 1 (Left), the position of a hypothetical unlabeled subject is marked by the blue *X*. Two projections are also calculated, corresponding to the expected value of the second bin given the value of the first, according to the control (green dotted line) and disease (red dotted line) models. These two values are used to construct the graph of Figure 1 (Right). Two normal distributions are plotted, centered on the expected values calculated in the previous step, and with widths equal to the standard deviation of the corresponding vectors of residuals (*ε* and *ε'*).

Taking into account the expected value of the second bin in both models and the corresponding expected error in the lineal fit (given by the standard deviation of residuals), the probability 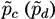 for subject *X* of pertaining to the control (patient) group is proportional to the value of the corresponding normal distribution at the point defined by the second bin. As *X* must be classified into one of the two classes, the final probability of pertaining to the patient class is given by the normalization:

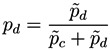
(2)


It must be noticed that the result of this process is a number, defined within the interval [0, 1], associated to a pair of measurements (or nodes of the network). In other words, we can construct the network of *n* nodes of subject *X* by analyzing the links associated to all possible *n* (*n* −1) pairs of nodes, to each of which is associated a weight defining how much the values of these two bins are close to the disease model. The final result is then a weighted network for each subject under study. 

## 3. Classification of Subjects

As a first application and example of the proposed network reconstruction algorithm, we consider a data set of metabolic spectral measurements, corresponding to 25 control subjects, and 25 patients suffering from *Glomerulonephritis* (*GN*). GN designates a group of renal diseases, characterized by an inflammation of glomerular capillaries, leading to a strong reduction in the renal function [17]. These data are a subset of the information considered in Refs. [18,19,20]. They include, for each subject of the two groups, a proton-NMR spectrum calculated from a urine sample; these spectra have been filtered by removing water regions and drug peaks, and subsequently binned into 200 bins of 0.04 ppm width. 

**Figure 2:**
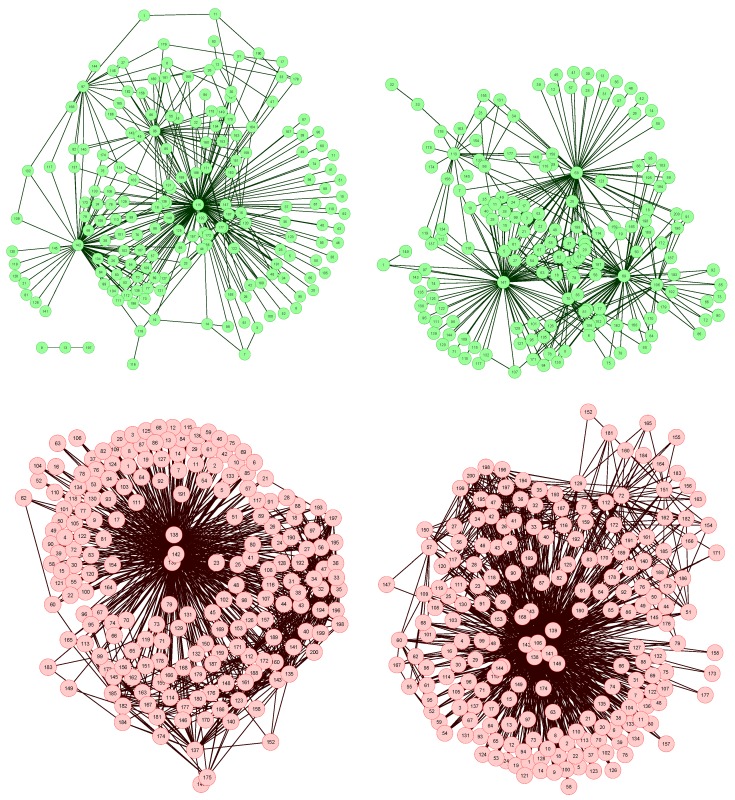
Four examples of network representation of spectral data. Upper (bottom) networks represent control subjects (patients suffering from Glomerulonephritis).

The network reconstruction algorithm previously described has been applied to such data set, each node representing a bin of the spectra. The resulting network representations for four subjects, two of them of the control group (upper part, in green) and two of the patient group (lower part, in red), are shown in Figure 2. In order to simplify the image, only links with weight higher than 0.65 are represented. 

Two features can be easily recognized. Firstly, the two networks of the control group have less links (lower link density) than the other two. This effect is to be expected, as data corresponding to GN patients should be closer to the disease model, as defined in Figure 1 (Left), and therefore the weight associated to their links should be higher. 

Secondly, while control subject networks lack a clear structure, in the GN networks there is one or a few nodes with a central position, *i.e*., concentrating most of the connections. This relationship between the network topology and the health condition of the subject is a consequence of the way the network is created. In the ideal case, for networks corresponding to control subjects, we would expect all links to have a zero weight, *i.e*., all data should perfectly fit the control model. Clearly, this is impossible, as biological systems are not perfect, and measurement errors may introduce further noise into the data. The consequence of this noise is that some links may gain a higher weight. Yet, if no pattern is present in the noise, these promoted links should form a random structure. On the other hand, when analyzing GN networks, the disease is expected to manifest mainly at some bins, indicating the presence (or absence) of specific metabolites. This implies that most links, *i.e*., relationships characteristic of the disease, should gather around these bins.

**Figure 3:**
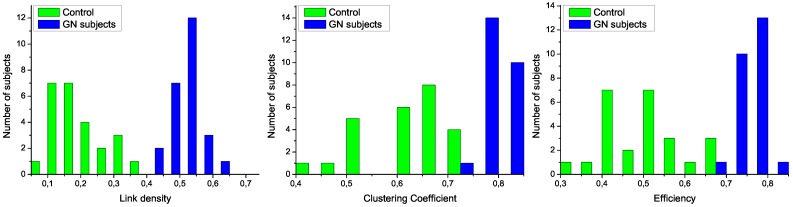
Analysis of the structural characteristics of networks for control subjects (green) and patients (blue). The three plots represent the histograms for (left) link density, (center) clustering coefficient, and (right) efficiency-see text for definitions.

To further analyze the differences between networks representing both groups of subjects, in Figure 3 are presented the histograms corresponding to several network metrics. Specifically, the metrics considered here are the following:

- *Link density*, simply defined as the number of expressed links, divided by the number of links that maybe presentinthe network [21]. Following the usual notation of network theory [8], the density is given by:

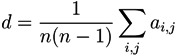
(3)
being *n* the number of nodes in the network and *a* its adjacency matrix, whose value *a_i,j_* is equal to one if there exists a link connecting nodes *i* and *j*, and zero otherwise.
- *Clustering Coefficient*, defined as the normalized number triangles present in the network [22]. A high clustering coefficient has been historically associated to social networks, where it means “the friends of my friends are also my friends”. Mathematically, it is given by:

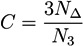
(4)
*N*_∆_ being the number of triangles in the network, and *N*_3_ the number of connected triples.
- *Efficiency*, assessing the performance of the network in transmitting information [23]. It is defined as the mean of the inverse of the lengths of the shortest paths connecting pairs of nodes:

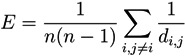
(5)
Here, *d_i,j_* represents the length of the shortest path connecting nodes *i* and *j*.

The structural features that have been manually identified in Figure 2 are now confirmed in the three histograms of Figure 3. Specifically, all networks associated to GN subjects have a higher link density, with the value of 0.4 being a natural threshold for the classification of both groups. Furthermore, the clustering coefficient and the efficiency are lower in control subjects, indicating a more random structure; on the contrary, the *star-like* topology associated with GN subjects has a very high efficiency, as most nodes are connected by a path of length 2. Such results indicate that control subjects and patients must respectively be associated to *Poisson-like* and *scale-free* degree distributions, two important families of graphs that have been extensively studied in the last decades [6,8]. 

**Figure 4:**
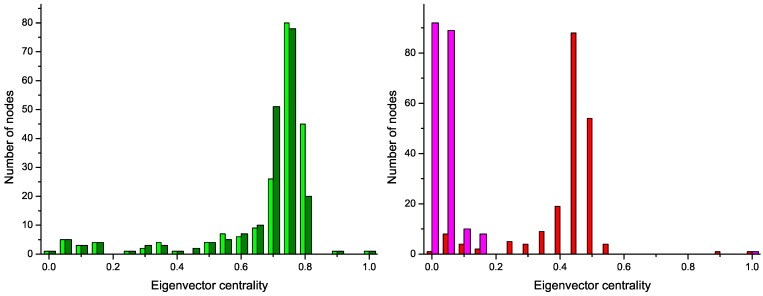
Histograms of the eigenvector centrality of nodes of networks represented in Figure 2, *i.e*., two control subjects (Left) and two GN patients (Right).

These two structures are confirmed by the analysis of the distribution of node centrality. *Centrality* is a general term that refers to the importance of a node in the network. Clearly, both in random graphs and regular lattices, each node is essentially equivalent to all other nodes, but when more complicated structures appear, one node may become especially important for the system. In this work we focus on the *eigenvector centrality*, which considers that a node has high importance if it is itself connected to other central positions [24]. Figure 4 reports the four centrality histograms, corresponding to the four networks depicted in Figure 2. It can be noticed that control subject networks are characterized by a flatter centrality distribution, in which all nodes have medium importance; on the other side, nodes in networks corresponding to GN patients have on average a low centrality, except for some highly important nodes.

The classification of subjects can be easily performed by any of the standard data mining algorithms available in the literature [25], by using the extracted network features (e.g., link density, efficiency, and so forth) as input in the process (see Ref. [26] for an analysis of this topic). By using *Support Vector Machines* [27] with link density and efficiency as input features, and a *leave-one-out* validation technique, a 100% score is easily achieved. It is worth noticing that perfect classification has been reached directly from the raw spectral data, without the use of any of the standard pre-processing techniques, like smoothing, baseline correction, or Principal Component Analysis [3]. Indeed, one of the advantages of this complex network approach is that noise, and other artifacts in the data, may locally affect the structure of the network, but they do not affect the global properties of the resulting structure.

Furthermore, the analysis of the most central nodes in each network provides valuable information about the bins defining the health status of the subject. As can be seen in Figure 2 (Bottom), the most important nodes are number 138, 139 and 142, corresponding to segments centered in *δ*^1^H 9.44–9.6 ppm (associated to CH and CHO signals). 

**Figure 5:**
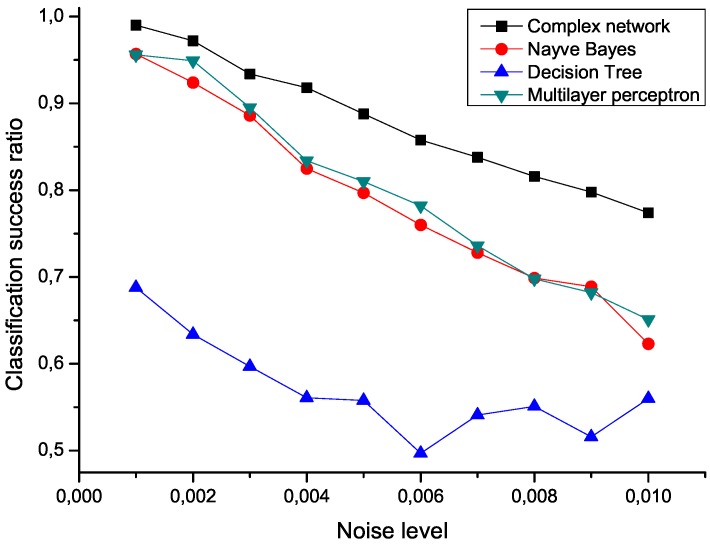
Mean classification scores obtained by four algorithms for data sets polluted with additive noise; the proposed network-based representation is represented by black squares.

To check the sensitivity of the proposed algorithm to the presence of noise, an ensemble of 100 modified data sets has been created, by polluting the original measurements with an additive noise drawn from a normal distribution center in zero. Figure 5 presents the mean classification score of several algorithms as a function of the standard deviation of the noise. The proposed network representation outperforms the other three considered classification techniques, *i.e*., naive Bayes, decision trees and multilayer perceptrons [25,28], thus showing a great robustness against noise contamination. 

## 4. Analysis of Disease Evolution

To show the wide range of applications in which the proposed network reconstruction algorithm is of help, we move from the previously described classification problem to the issue of monitoring the evolution of the status of a patient through time. 

The data set analyzed has been constructed by collecting Raman spectra [29,30] from 133 blood samples, 102 of them corresponding to control subjects, and 31 to persons suffering from *leukemia*. A Raman spectrum is a fingerprint of biological sample, and its bands provide information about the conformation of macromolecules, such as proteins, nucleic acids, or lipids. Control samples correspond to people who presented themselves as potentially blood donors at the *Hospital Regional de Alta Especialidad del Baj´ıo* (HRAEB), Guanajuato, Mexico; data include both male and female with a mean age of 25, who had no alcohol nor drugs in the last 72 hours. The second group comprises people suffering from three different types of leukemia, *Acute Lymphoblastic Leukemia* (ALL), *Acute Myeloid Leukemia* (AML) and *Chronic Myelogenous Leukemia* (CML), with ages spanning from 5 to 80 years. In all cases, blood samples were centrifuged in order to separate the plasma and then analyzed using a *Horiba Jobin Yvon LabRAM HR800* micro-Raman System. Samples were radiated with an 830 nm laser diode of 17 mW power, and the resulting spectra were recorded with an 800 mm focal length spectrograph. Written consent was obtained from the subjects and the study was conducted according to the Declaration of Helsinki.

**Figure 6:**
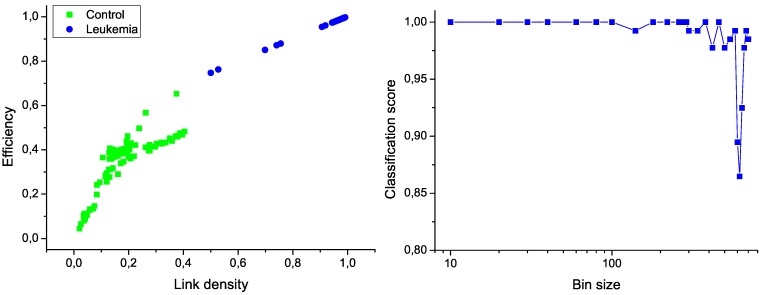
Classification of control and leukemia subjects. (Left) Representation of the position of control subjects (green squares) and leukemia patients (blue points) in the space of network features. (Right) Classification score as a function of the binning size.

As a first step, both sets of data, corresponding to control subjects and patients, have been used to train the model, *i.e*., to calculate the regression lines as in Figure 1. The same set of subjects have then been classified using a Support Vector Machine [27] algorithm and *leave-one-out* validation technique, and the feature selection method proposed in Ref. [26]. In order to reduce the computational cost, the original measurements have been binned by using different widths. The two most relevant network characteristics for this task, corresponding to a bin size of 10 measurements, are shown in Figure 6 (left); the clear separation between the two groups graphically confirms the classification score of 100%. Additionally, Figure 6 (right) presents the evolution of the classification score as a function of the bin size; results indicate that the classification is robust, and that the task score lowers only when the number of nodes in the network is drastically reduced. 

**Figure 7:**
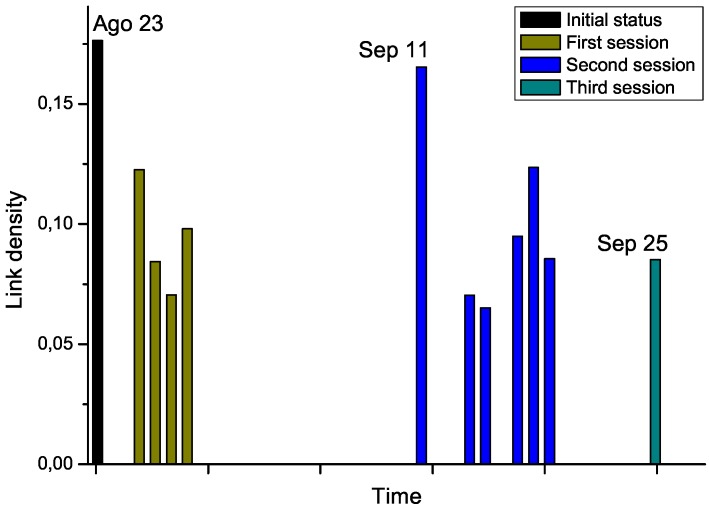
Evolution through time of the link density of networks representing a leukemia patient under chemotherapy treatment.

For the second phase, we got access to the Raman spectra corresponding to an additional patient, who was diagnosed with leukemia and underwent chemotherapy treatment. Notably, several measurements were available, for the day before the start of the therapy, and for each treatment days (*i.e*., after the chemotherapy sessions), grouped in three sessions. By analyzing the Raman spectra after each chemotherapy session, this time-dependent information allows us tracking the evolution through time of the structure of the network. The results corresponding to link density are reported in Figure 7. 

Chemotherapy seems to globally lower the link density of the network, therefore representing an improvement in the status of the patient. Nevertheless, the network corresponding to September 11^*th*^, which has been measured after a long pause of two weeks in the treatment, suggests a return to the initial status. While no other biomedical information was available for this subject, and thus no significant conclusions can be drawn from this example, Figure 7 suggests that the proposed network representation of spectral data may be used for tracking patient status, providing a new tool for decision making processes in different treatments, as suggested in Ref. [30]. For instance, the doctor may have decided to anticipate the start of the second session to avoid relapses. 

## 5. Conclusions

In this contribution, we proposed a method based on complex network theory for the discrimination of pathological patterns in metabolic spectra associated with different physiological samples (urine and blood of patients suffering respectively from *Glomerulonephritis* and leukemia). In this approach alternative to standard supervised data mining techniques [3,4,5], each subject is associated with a network whose nodes represent different data bins, and the links between these nodes are established when the relationship between their values follows the behavior expected in the disease and extracted from labeled data. The graph-theoretical metrics [21] of the resulting network are ultimately used to train standard data mining algorithm models, representing the whole data set. The models are then used to perform different knowledge discovery tasks. 

Our results show that the network structures of control subjects and patients are significantly different, and that this difference can be used to achieve a 100% classification score using Support Vector Machines and *leave-one-out* validation scheme.

The proposed method offers clear advantages in terms of relevant knowledge extraction from data:

- When analyzing a network structure, the addition or deletion of a single link has minimal effects on the overall topology. Therefore, data mining results obtained from features representing such topologies are robust to the presence of noise in the initial data set. Furthermore, the whole initial data set, which may be composed of thousands of individual measurements, is reduced to a few features. Therefore, *pre-processing steps, like feature selection or noise reduction, are not required*.
- *The network structure highlights in a very visual way the elements that are relevant for the identification of control subjects and patients* (as in Figure 2); therefore, the proposed methodology allows a simple and intuitive interpretation of results.
- The information relative to a subject is synthesized into one or a few features, and *it is possible to quantitatively track the evolution of a disease through time*. This opens new possibilities for monitoring complex treatments.

Although further analyses are needed to corroborate and validate these last three points, we expect the proposed methodology to offer new avenues for the solution of problems in metabolomics, such as metabolomic fingerprinting and profiling. 
